# Two-stage posterior spinal fusion for early-onset scoliosis: Two case reports

**DOI:** 10.1097/MD.0000000000014728

**Published:** 2019-03-01

**Authors:** Masashi Uehara, Jun Takahashi, Shugo Kuraishi, Shota Ikegami, Toshimasa Futatsugi, Hiroki Oba, Takashi Takizawa, Ryo Munakata, Michihiko Koseki, Hiroyuki Kato

**Affiliations:** Department of Orthopaedic Surgery, Shinshu University School of Medicine, Matsumoto, Nagano, Japan.

**Keywords:** early-onset scoliosis, posterior spinal fusion, radiological findings, T1-T12 length, 2-stage surgery

## Abstract

**Rationale:**

Fusionless techniques for early-onset scoliosis (EOS) have evolved to allow near-normal growth while maintaining the correction achieved during the initial surgery. However, such procedures require repeated surgeries and have increased complication rates. We have developed a 2-stage fusion technique using pedicle screws for EOS to reduce patient burden and complication risk. This series describes the clinical and radiological features of 2 patients with EOS who received 2-stage posterior spinal fusion. This surgical method for EOS represents the first of its kind.

**Patient concerns:**

Case 1 was a 10-year-old girl who was diagnosed as having scoliosis with Prader Willi syndrome at the age of 2 years. Her preoperative major curve Cobb angle was 100 degrees at age 10 years. Case 2 was an 11-year-old boy who was found to have scoliosis with 22q11.2 deletion syndrome at the age of 4 years. His preoperative major curve Cobb angle was 77 degrees at age 11 years.

**Diagnosis:**

Whole-spine radiographs were performed to diagnose scoliosis.

**Interventions:**

Both patients received 2-stage posterior spinal fusion.

**Outcomes:**

Postoperative Cobb angle of the major curve improved to 46 and 48 degrees, respectively. Thoracic height respectively improved from 160 and 148 mm before surgery to 206 and 211 mm at final follow-up. Surgical outcome as evaluated by Scoliosis Research Society-22 patient questionnaires revealed acceptable results without any severe complications.

**Lessons:**

Based on the present case report, 2-stage posterior spinal fusion for EOS achieves good radiological and clinical outcomes without severe complications.

## 1 Introduction

Surgical treatment for early-onset scoliosis (EOS) has always been a challenge for spine surgeons. The goal of EOS management is to control the spinal deformity without interfering with spinal growth,^[1–3]^ but early definitive fusion before the age of 10 years may not prevent deformity progression and can cause a crankshaft phenomenon^[4,5]^ and/or thoracic insufficiency syndrome.^[6–8]^

Moe et al^[9]^ first described the distraction-based growing rod (GR) system in 1984. Fusionless techniques, including GRs and vertical expandable prosthetic titanium rib devices, have evolved to allow near-normal growth while maintaining the correction achieved during the initial surgery. However, repeated surgeries are needed every 6 to 9 months on average to allow the spine and chest to grow.^[10–13]^ Of particular concern are significant complication rates, increased costs due to planned and unplanned procedures, and psychological consequences.^[14–16]^ DiMaggio et al identified a modestly elevated risk of adverse behavioral or developmental outcomes in children who were exposed to anesthesia during early childhood based on existent epidemiologic evidence.^[17]^

We have been developing 2-stage surgery for posterior spinal fixation of EOS in order to reduce patient burden and the risk of complications. The present series describes the clinical and radiological features of 2 patients with EOS who received 2-stage posterior spinal fusion. To the best of our knowledge, this surgical method for EOS represents the first of its kind.

## 2 Case reports

This was a retrospective case series study that included 2 patients with EOS aged 10 and 11 years, respectively, at the first surgery who were treated by 2-stage posterior spinal fusion using pedicle screws. Pedicle screw insertion was performed using a CT-based navigation system (Stealth Station 7; Medtronic, Sofamor Danek, Memphis, TN). Scoliosis was diagnosed at 2 and 4 years of age, respectively. Primary disease was syndromic scoliosis (Prader Willi syndrome and 22q11.2 deletion syndrome) in 2 patients. Written informed consent was obtained from the patients’ parent for publication, including any necessary photographs. This surgical method was approved by the ethics committee of our hospital (No. 1713).

The respective follow-up periods were 80 and 71 months after the first operation (posterior short fusion between end vertebrae of the main curve) and 33 and 24 months after the second operation (posterior spinal fusion). The cohort's demographic data are summarized in Table 1. Pre- and postoperative examination and surgical data are presented in Tables 2–4.

**Table 1:**
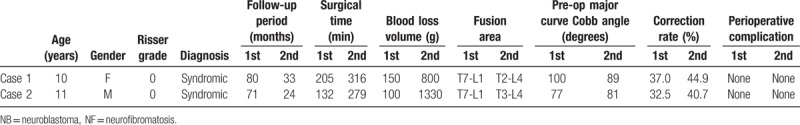
Demographic data.

**Table 2:**
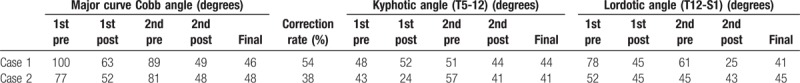
Pre- and postoperative examination and surgical data (1).

**Table 3:**
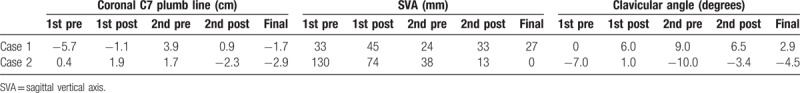
Pre- and postoperative examination and surgical data (2).

**Table 4:**
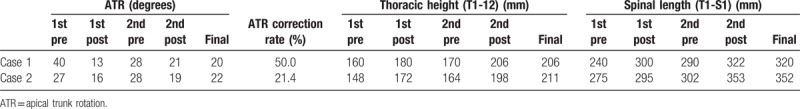
Pre- and postoperative examination and surgical data (3).

Radiographs were evaluated to assess the height of the thoracic spine, defined as the vertical distance between T1 and T12. Thoracic height improved from 160 and 148 mm before surgery to 206 and 211 mm at final follow-up, respectively. Surgical outcome as evaluated by Scoliosis Research Society (SRS)-22 questionnaires revealed favorable results without severe complications (Table 5). No surgical site infection or implant-related complications were detected during the treatment period.

**Table 5:**
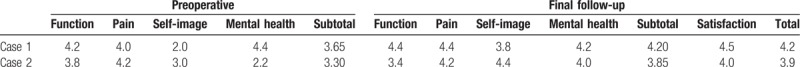
Scoliosis Research Society-22 patient questionnaire results.

**Figure 1:**
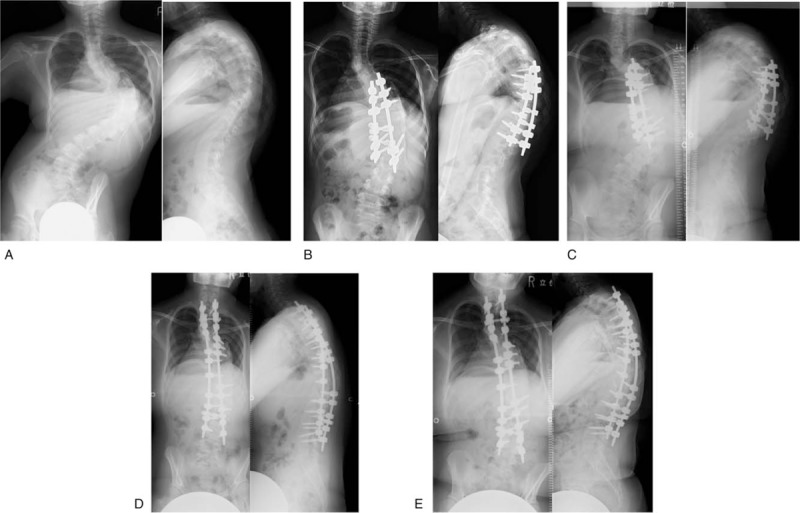
Case 1: a 10-year-old girl with Prader Willi syndrome. (A) Preoperative Cobb angle of the major curve was 100 degrees. (B) We performed stage 1 posterior spinal fusion from T7 to L1. Postoperative Cobb angle of the major curve improved to 63 degrees. (C) Cobb angle of the major curve was 89 degrees before the second surgery. (D) We performed stage 2 posterior spinal fusion from T2 to L4. Postoperative Cobb angle of the major curve improved to 49 degrees. (E) The major curve Cobb angle was maintained at 46 degrees at the final follow-up.

Case 1: A 10-year-old girl was diagnosed as having scoliosis with Prader Willi syndrome at the age of 2 years. She received brace treatment from 5 to 10 years of age. We performed stage 1 posterior short fusion from T7 to L1 for her preoperative major curve Cobb angle of 100 degrees and angle of trunk rotation (ATR) of 40 degrees at the age of 10 years. Preoperative height was 117 cm, weight was 25.3 kg, and body mass index (BMI) was 18.5 kg/m^2^. Her height was equivalent to that of a child of 6 years and 8 months. Although, Y cartilage was closed, Risser grade was 0 and she was before the first menstruation before the first surgery. So, we judged that there was a possibility of growing yet, and 2 staged surgery was applied. Surgical time was 205 minutes and blood loss volume was 150 g. Postoperative Cobb angle of the major curve improved to 63 degrees. Postoperative ATR improved to 13 degrees. Postoperative brace therapy was continued to prevent a crank shaft phenomenon. We performed stage 2 posterior spinal fusion from T2 to L4 for her preoperative major curve Cobb angle of 89 degrees and ATR of 28 degrees at the age of 14 years. Preoperative height was 122 cm, weight was 38.8 kg, and BMI was 26.0 kg/m^2^. Her height was equivalent to that of a child of 7 years and 7 months. Risser grade was 3 before the second surgery. Surgical time was 316 minutes and blood loss volume was 800 g. Postoperative Cobb angle of the major curve improved to 46 degrees. Postoperative ATR improved to 20 degrees. Thoracic height (T1-12) had improved from 160 to 206 mm and spinal length (T1-S1) had increased from 240 to 320 mm at the final follow-up (Fig. 1). Forced vital capacity improved from 0.5 L before surgery to 0.88 L afterwards. Preoperative SRS-22 domain scores were 4.2, 4.0, 2.0, 4.4, and 3.65 for function, pain, self-image, mental health, and subtotal, respectively, which were ameliorated at the final follow-up at 4.0, 4.6, 2.8, 4.2, and 3.9, respectively. There were no severe perioperative complications.

**Figure 2:**
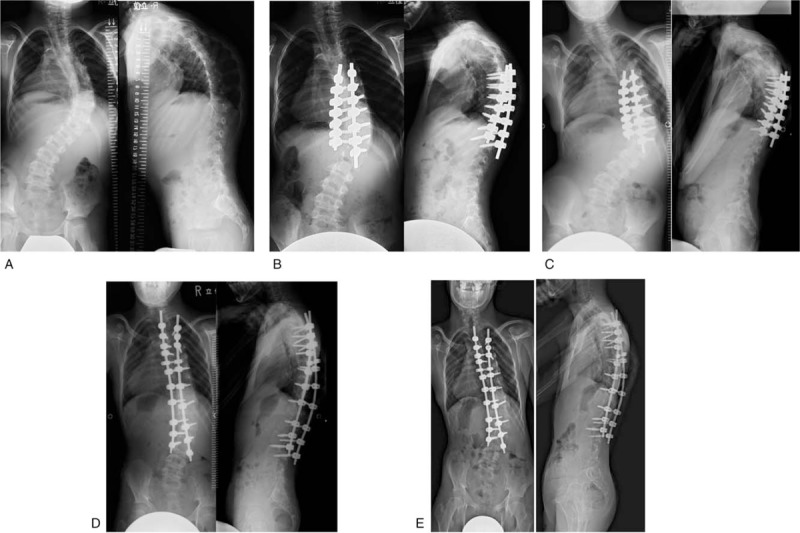
Case 2: an 11-year-old boy with 22q11.2 deletion syndrome. (A) Preoperative Cobb angle of the major curve was 77 degrees. (B) We performed stage 1 posterior spinal fusion from T7 to L1. Postoperative Cobb angle of the major curve improved to 52 degrees. (C) Cobb angle of the major curve was 81 degrees before the second surgery. (D) We performed stage 2 posterior spinal fusion from T3 to L4. Postoperative Cobb angle of the major curve improved to 48 degrees. (E) The major curve Cobb angle was maintained at 48 degrees at the final follow-up.

Case 2: An 11-year-old boy was diagnosed as having scoliosis with 22q11.2 deletion syndrome at the age of 4 years. He received brace treatment from 4 to 11 years of age. We performed stage 1 posterior short fusion from T7 to L1 for his preoperative major curve Cobb angle of 77 degrees and ATR of 27 degrees at the age of 11 years. Preoperative height was 114 cm, weight was 17.0 kg, and BMI was 13.1 kg/m^2^. His height was equivalent to that of a child of 6 years and 1 month. Risser grade was 0 before the first surgery. Surgical time was 132 minutes and blood loss volume was 100 g. Postoperative Cobb angle of the major curve improved to 52 degrees. Postoperative ATR improved to 16 degrees. Postoperative brace treatment was continued to prevent a crank shaft phenomenon. We performed stage 2 posterior spinal fusion from T3 to L4 for his preoperative major curve Cobb angle of 81 degrees and ATR of 28 degrees at the age of 15 years. Preoperative height was 123 cm, weight 24.6 was kg, and BMI was 16.2 kg/m^2^. His height was equivalent to that of a child of 7 years and 7 months. Risser grade was 0 before the second surgery. Surgical time was 279 minutes and blood loss volume was 1330 g. Postoperative Cobb angle of the major curve improved to 48 degrees. Postoperative ATR improved to 22 degrees. Thoracic height had improved from 148 to 211 mm and spinal length had increased from 275 to 352 mm at the final follow-up (Fig. 2). Forced vital capacity improved from 0.38 L before surgery to 0.62 L afterwards. Preoperative SRS-22 domain scores were 3.8, 4.2, 3.0, 2.2, and 3.3 for function, pain, self-image, mental health, and subtotal, respectively, which were improved at the final follow-up at 3.4, 4.2, 4.4, 3.4, and 3.85, respectively. There were no severe perioperative complications.

## 3 Discussion

In the present case series of 2 patients with EOS receiving 2-stage posterior spinal fusion for severe scoliosis, pedicle screw fixation improved radiological parameters, clinical findings, and respiratory function without severe perioperative complications. The concept of this technique is to secure the growth of the patient's spinal length and to avoid multiple operations while allowing some amount of the occurrence of crankshaft phenomenon. Currently, we apply this method to cases with a Cobb angle of 70 degrees or more in the supine position.

EOS designates patients under 10 years of age who have a spinal deformity with structural curve. Normal spinal fusion surgery is not indicated in these cases since the skeleton is immature. If left untreated, however, EOS results in a high degree of curvature combined with thoracic deformity that may lead to pulmonary hypoplasia and respiratory disturbance. Fusionless techniques permit near-normal growth while maintaining good correction but require repeated surgeries that may place considerable burden on the patient.^[10–16]^ Bess et al reported that the incidence of complications increased by 24% with each rod extension operation and that 58% of patients experienced at least 1 complication.^[18]^ In a systematic review of a magnetically controlled GR, the mean complication rate was 44.5% and unplanned revision rate was 33%.^[19]^ Furthermore, Poe-Kochert et al reported many reoperations even after final fusion in GR treatment.^[20]^ In our series, no perioperative complications were observed, such as surgical site infection or severe adverse implant-related effects. The main advantage of this method is that it is completed in only 2 operations.

When aggressive posterior fusion is performed on immature skeletal patients, there is concern of a crankshaft phenomenon afterwards since the anterior column continues to grow.^[5]^ Although underarm brace treatment was continued in our 2 cases after the first surgery to prevent a postoperative crank shaft phenomenon, ATR increased from 13 and 16 degrees immediately after surgery to 28 and 28 degrees, respectively, before the second operation at 47 months. However, the hump was corrected in the second surgery and ATR was respectively improved at 20 and 22 degrees at the final follow-up. The respective ATR correction rates were 50.0 and 21.4%.

Regarding treatment with GR, a study of 13 patients followed until the final fixation (mean: 5.7 years) demonstrated that Cobb angle improved from 81 to 27.7 degrees and that T1-S1 spinal length was increased by an average of 5.7 cm.^[12]^ In our series, major curve Cobb angle improved from 100 and 77 degrees to 46 and 48 degrees and spinal length was increased by 8.0 and 7.8 cm at the final follow-up (follow-up period: 6.7 and 5.9 years), respectively. Compared with Akbarnia report on GRs, the correction rate of 2-stage posterior spinal fusion was inferior but spinal length improvement was good. Luhmann et al reported that the Shilla Growth Guidance System (SGGS) extended by 46 mm (7.5 mm per year) for T1-T12 in 6.1 years and 52 mm (7 mm per year) for traditional GR treatment in 7.4 years.^[21]^ Our procedure extended spinal length by 46 and 63 mm (6.9 and 10.7 mm per year) at 80 and 71 months, respectively. Compared with SGGS, GRs have nearly twice the elongation of the thorax, and the elongation of the trunk indicates the performance of this procedure is good. The mean correction rates in our study were 54% and 38% and comparable to those of SGGS and GR (44% and 45%, respectively). Although the correction rate in this case series was lower than in a previous adolescent idiopathic scoliosis series (46.0% vs 62.4%^[22]^), surgical outcome as evaluated by SRS-22 showed acceptable results without severe complications. Perhaps we should have added Ponte osteotomy to the fusion mass during the second surgery to increase the final correction rate. Nonetheless, sagittal vertical axis was improved from 33 and 130 mm to 27 and 0 mm, respectively.

Thoracic insufficiency syndrome described by Campbell et al provides compelling evidence for EOS treatment intervention.^[23]^ Thoracic height, which is an indicator of the growth of the thorax, is ideally greater than 22 cm.^[24]^ In our study, thoracic height improved from 16.0 and 14.8 cm before surgery to 20.6 and 21.1 cm at final follow-up, respectively. Average forced vital capacity was improved in both patients.

Lastly, although our 2-stage technique was effective both radiologically and clinically, the initial surgery was performed on patients 10 or more years old. However, cases 1 and 2 had developmental disorders and their age-adjusted heights were 7.6 and 6.1 years, respectively, and the average height of the 2 cases was equivalent to that of a child of 7 years. Further study is needed on EOS patients under 10 years of age who require surgery.

The limitations of this case report are a small sample and short-term follow-up. Moreover, a learning curve exists to insert screws into the thin pedicle roots on the concave side of the apical vertebra. Familiarity with navigational surgery if advised. The selection recommendations for this technique are pediatric patients of relatively high age with EOS.

In conclusion, based on the present case series, 2-stage posterior spinal fusion for EOS achieves good radiological and clinical outcomes without severe complications. This procedure can be an option of treatment for EOS.

## Author contributions

**Data curation:** Masashi Uehara, Jun Takahashi, Shota Ikegami, Shugo Kuraishi, Toshimasa Futatsugi, Hiroki Oba, Takashi Takizawa, Ryo Munakata, Michihiko Koseki, Hiroyuki Kato.

**Formal analysis:** Masashi Uehara, Hiroyuki Kato.

**Investigation:** Masashi Uehara, Jun Takahashi, Shota Ikegami, Shugo Kuraishi, Hiroki Oba, Takashi Takizawa, Ryo Munakata, Michihiko Koseki.

**Methodology:** Jun Takahashi, Shota Ikegami, Shugo Kuraishi.

**Supervision:** Jun Takahashi, Michihiko Koseki, Hiroyuki Kato.

**Writing – original draft:** Masashi Uehara.

**Writing – review & editing:** Jun Takahashi, Shota Ikegami, Shugo Kuraishi, Toshimasa Futatsugi, Hiroki Oba, Takashi Takizawa, Ryo Munakata, Michihiko Koseki, Hiroyuki Kato.
